# Development of a Point-of-Care Assay for HIV-1 Viral Load Using Higher Refractive Index Antibody-Coated Microbeads

**DOI:** 10.3390/s21051819

**Published:** 2021-03-05

**Authors:** Mazhar Sher, Benjamin Coleman, Massimo Caputi, Waseem Asghar

**Affiliations:** 1 Asghar-Lab, Micro and Nanotechnology in Medicine, College of Engineering and Computer Science, Boca Raton, FL 33431, USA; msher2015@fau.edu; 2 Department of Computer & Electrical Engineering and Computer Science, Florida Atlantic University, Boca Raton, FL 33431, USA; 3 Department of Electrical and Computer Engineering, Rice University, 6100 Main Street, Houston, TX 77005, USA; ben.coleman@rice.edu; 4 Charles E. Schmidt College of Medicine, Florida Atlantic University, Boca Raton, FL 33431, USA; mcaputi@fau.edu; 5 Department of Biological Sciences (Courtesy Appointment), Florida Atlantic University, Boca Raton, FL 33431, USA

**Keywords:** HIV-1, point-of-care diagnosis, lensless imaging, computational analysis, portable systems

## Abstract

The detection of viruses using imaging techniques is challenging because of the weak scattering of light generated by the targets of sizes in the nanometer range. The system we have developed overcomes the light scattering problems by utilizing antibody-coated microbeads of higher index of refraction that can specifically bind with viruses and increase the acceptance angle. Using the new technology, we have developed a portable, cost-effective, and field-deployable platform for the rapid quantification of HIV-1 viral load for point-of-care (POC) settings. The system combines microfluidics with a wide field of view lensless imaging technology. Highly specific antibodies are functionalized to a glass slide inside a microchip to capture HIV-1 virions. The captured virions are then bound by antibody-conjugated microbeads, which have a higher refraction index. The microbeads—HIV-1 virions complexes generate diffraction patterns that are detected with a custom-built imaging setup and rapidly and accurately quantified by computational analysis. This platform technology enables fast nanoscale virus imaging and quantification from biological samples and thus can play a significant role in the detection and management of viral diseases.

## 1. Introduction

Human Immunodeficiency Virus type I (HIV-1), the causative agent for AIDS, is still considered a global healthcare threat, having claimed more than 32 million human lives since the start of the epidemic through the end of 2018 and currently affecting an estimated 38 million people worldwide [1,2]. Minimizing the spread of this virus and reducing its mortality are dependent on the identification of the viral infection at an early stage and continuous access to treatment and diagnostics facilities to evaluate the viral load in patients. Antiretroviral therapy (ART) has been proven to be successful in reducing the mortality associated with HIV-1/AIDS and keeping the viral load under control [3,4,5,6,7]. The viral load is utilized to monitor the patient’s response to ART to ensure drug adherence and prevent the emergence of resistance. Since this disease is prevalent in resource-limited areas, it is paramount to develop simple, cost-effective, and user-friendly devices that can enable early-stage HIV-1 detection and viral load quantification. Early-stage diagnosis can also help to quickly initiate the treatment and reduce the viral load to a suppressed state before a high viremia and viral spread are established [8]. Thus, helping in disease management and outcome while significantly reducing further transmission of the virus in the population. The current gold standard for viral load measurement is based on reverse-transcriptase-quantitative polymerase chain reaction (RT-qPCR) [9]. This nucleic acid-based amplification method utilizes expensive equipment, several reagents, and skilled trained professionals, which are required to conduct the test and analyze the results. Overall, RT-qPCR is a labor-intensive, time-consuming, and technically complex process [10] and is therefore not suitable for point of care (POC) and resource-constrained settings. Recent advances in the field of microfluidics have significantly contributed to viral diagnostics [11]. A portable microchip that incorporates magnetic beads conjugated with an anti-HIV1 biotinylated antibody can be utilized to capture HIV-1 virions from plasma samples [12] and quantify the captured virions using electrical impedance spectroscopy. Unfortunately, electrical impedance-based virus detection exhibits low sensitivity. Alternatively, microchips can be functionalized with highly specific antibodies to capture the virus from various types of bodily samples. The captured HIV-1 particles can be quantified using quantum dots [13]. However, this technique relies on the utilization of an expensive fluorescence microscope with a limited field of view, greatly limiting the application of this method in POC settings. Previous efforts to detect viruses using imaging setups [14,15] indicated that the weak light scattering and interaction with nanoscale virions makes it very difficult to image virions directly from the sample. A lower refractive index contrast to the surrounding medium, and weak interaction with photons further complicate direct optical detection [16,17]. Electron microscopy is routinely utilized to image viruses [18,19,20,21,22,23,24]. However, this technique provides a limited field of view, is labor-intensive and extremely expensive; hence it is not suitable for POC settings.

Here, we present a new cost-effective method for the quantification of HIV-1 viral particles that utilizes a surface-functionalized microchip, antibody-coated magnetic beads, a portable lensless imaging setup, and computational analysis software. Our method leverages functionalized microfluidic chip surfaces and high refractive index magnetic microbeads to quantify the HIV-1 viral load of biological samples. First, a microfluidic chip is functionalized with highly specific antibodies and utilized to capture the viral particles. Anti-HIV1 gp120 antibody-coated microbeads are then bound to the virions captured on the microchips. The diffraction patterns of the microbeads are then recorded using a Complementary Metal–Oxide–Semiconductor (CMOS) image sensor. Finally, a custom-made software is used to detect the captured microbeads and separate them from debris to perform the final viral load quantification.

## 2. Materials and Methods

### 2.1. Materials

Optically clear 76 microns thick double-sided adhesive (DSA) tape and 3.125 mm thick polymethyl methacrylate, (PMMA) were obtained from 3M (St. Paul, MN, USA) and McMaster-Carr (Atlanta, GA, USA), respectively. Bovine serum albumin (BSA) was purchased from Fisher Scientific (Fair Lawn, NJ, USA). A 10 mL syringe was purchased from Becton, Dickson and Company (Franklin Lake, NJ, USA). A blunt needle (17 gauge) from SAI (Lake Villa, IL, USA) was attached to 0.90″ outside diameter (OD) tube from Cole-Parmer (Vernon Hills, IL, USA) was connected to the syringe. This syringe was placed on the syringe pump purchased from New Era Pump Systems (East Farmingdale, NY, USA). Goat polyclonal antibody to HIV1 gp120 (biotin) catalog # ab53937 and goat polyclonal antibody HIV1 gp120 catalog # ab85054 were acquired from Abcam (Cambridge, MA, USA). Dynabeads™ Streptavidin Trial Kit Catalog # 65801D was purchased from Thermo Fisher (Waltham, MA, USA). Lipofectamine 2000, having catalog # 11668019, was obtained from Fisher Scientific (Waltham, MA, USA). Opti-MEM™ I Reduced Serum Medium, Catalog number# 31985062, was purchased from Gibco (city, state abbrev if USA, country). RT-PCR grade water with catalog # AM9935 was purchased from Fisher Scientific.

### 2.2. Methods

#### 2.2.1. Design and Fabrication of Portable Lensless Imaging Setup

We have designed and assembled a portable imaging platform having a large field of view (28.29 mm^2^). The lensless imaging setup consists of three main components:

(1) 385 nm light-emitting diode (LED) and an adjustable power supply.
(2) 100-micron pinhole.
(3) 18-megapixel CMOS image sensor (UI-3592LE).

**Figure 1:**
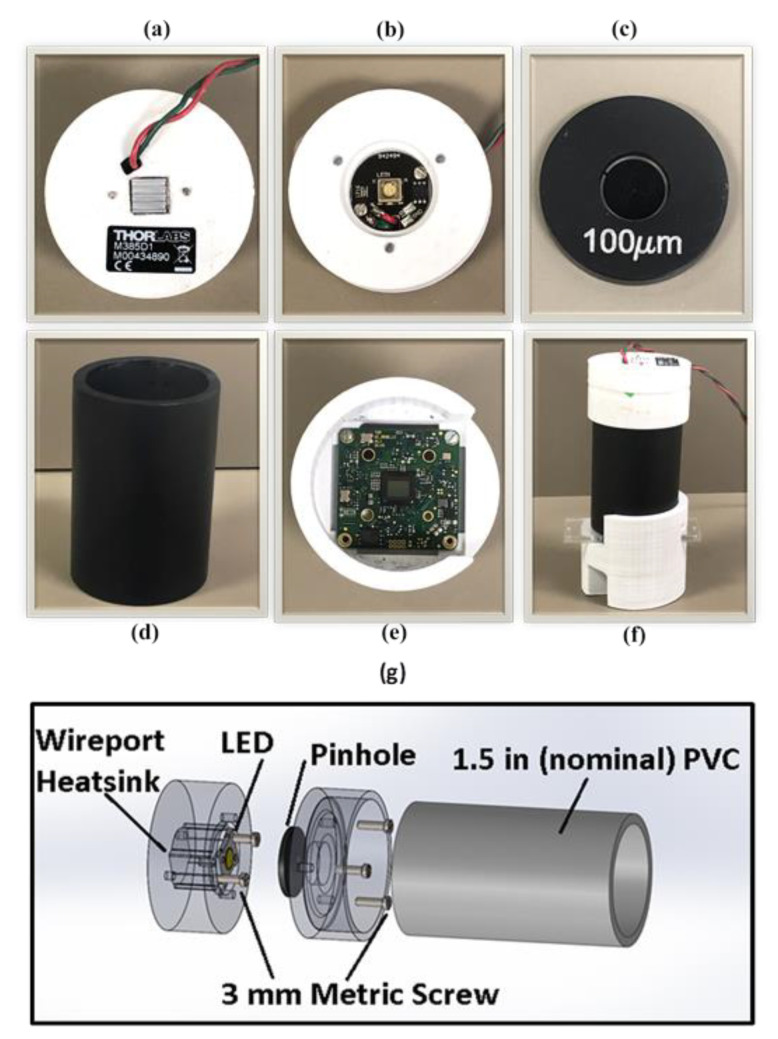
Components utilized in the development of portable imaging setup. (**a**) The 3D printed component containing the heat sink (**b**) LED fitted inside the custom-built housing. (**c**) 100 microns sized pinhole (**d**) PVC pipe (**e**) CMOS sensor mounted on a 3D printed base part (**f**) The complete lensless imaging setup with an antibody-coated microfluidic chip. (**g**) 3D sketch of the mechanical design of developed lensless imaging setup.

The particle detection process requires narrowband plane wave illumination. Both the wavelength and the propagation characteristics of the light source are critical for our image processing algorithms. As a result, the imaging platform is designed to provide illumination conditions that are sufficiently close to the ideal conditions without substantially increasing the cost of the device. The device is built to illuminate microfluidic chips with approximate plane waves at a 385 nm wavelength. The light is generated using a narrowband LED whose intensity can be easily tuned using an adjustable power supply. We pass the LED light through a small pinhole to create a light source that is approximately an ideal point source. At a sufficiently far distance, the radiation of a point source approximates that of an ideal plane wave. To create an approximate plane wave for imaging purposes, we separate the LED and pinhole from the image sensor using a long PVC tube. The tube is painted black to absorb reflections, suppress noise, and ensure that the light propagates correctly. At the end of the imaging chamber, a CMOS image sensor is utilized to record the diffraction patterns of captured microbeads. The developed lensless imaging platform and its components are shown in Figure 1.

The imaging system has the following characteristics:

- Field of View (FOV) =6.14 mm × 4.604 mm,
- Pixel size = 1.25-micron
- Overall dimensions = 60 mm × 60 mm × 138 mm (length × width × height)

Table S1 provides detailed information about the overall cost of the lensless imaging setup. The lensless imaging platform costs $1112.52, while the per-test cost is $14.062. The cost of commercially purchased antibody (Goat polyclonal to HIV1 gp120 (ab85054) was $13.66. The per-test cost can be significantly reduced by producing this antibody on a large scale. Prakash et al. investigated the cost of point-of-care viral load tests in two large clinics in Lilongwe, Malawi [25]. Both clinics were equipped with GeneXpert platform with four modules (Cepheid, Sunnyvale, CA, USA). The Xpert HIV-1 Viral load test using this platform provided automatic quantification of HIV viral load from 1 mL plasma sample in 104 min. The per-test cost was $33.71. The international benchmark for a centralized viral load test is $28.62. The POC GeneXpert device costs around $17,000.

#### 2.2.2. Microchip Fabrication

A microfluidic chip was assembled using the PMMA, DSA, and glass slide using previously published methods [26,27,28,29,30,31,32,33,34,35]. The chip design was made in AutoCAD 2015 from Autodesk, Inc. (San Rafael, CA, USA) and uploaded to the UCP software. VLS 2.30 CO_2_ laser cutter (Universal Laser Systems, Scottsdale, AZ, USA) was used to cut the design as per specifications. In each microfluidic device, two parallel channels (dimensions: 40 mm × 5 mm × 76 μm) were cut in DSA. One side of the DSA film was attached to PMMA, whereas the other side was attached to the antibody-coated glass slide. Each microchannel has one inlet and one outlet. Each microchannel can hold approximately 15 μL volume. As our lensless imaging setup has a field of view of only (6.14 mm × 4.604 mm), so, we devised a strategy to image the various subsections of the microfluidic channel. Consecutive demarcations were engraved on the top PMMA layers of the microchannel. Each demarcation resulted in an area of 20 mm^2^ (5 mm × 4 mm). This process ensured the reliable imaging of the whole microchannel. Table S1 presents information about the cost of materials utilized to assemble the microfluidic chip.

#### 2.2.3. Functionalization of Antibodies to Glass Slide

Glass slides were cleaned with 70% ethanol and dried using nitrogen gas. In order to form hydroxyl groups, these cleaned slides were treated with air plasma for 5 min. Then, 10 mg/mL thiol-PEG-silane (SH-PEG-Si) in 95% ethanol was immediately incubated on these slides for 30 min. After this step, washing was carried out with 70% ethanol. A cross-linker 3-[2-pyridyldithio] propionyl hydrazide (PI22301, Thermo-Fisher Scientific) with a concentration of 1 mg/ mL was incubated for 2 h in the dark at room temperature. The glass slides were cleaned with 70% ethanol and allowed to dry at room temperature. Microfluidic chips were assembled using the PMMA, DSA, and glass slides. 1 Molar solution of sodium acetate was made using nanopure water. The pH of this sodium acetate solution was adjusted to 5.5 using glacial acetic acid. Oxidation of the antibody was performed by mixing antibody with 10 mM sodium meta-periodate and 0.1 M sodium acetate (with pH equals to 5.5). The resulting solution was incubated in the dark at 4 °C for 30 min. The resulting oxidized antibody was incubated inside the microchip for one hour at room temperature. 4% BSA in PBS was utilized to block the unoccupied sites on the microchip. The blocked microfluidic chips were incubated overnight at 4 °C. After this step, the fully functionalized microfluidic chips were immediately used for the biological assay. The overall cost of reagents utilized in this antibody functionalization process is provided in Table S1.

#### 2.2.4. Viral Sample Preparation

Human Embryonic Kidney T cells (HEK 293T) purchased from American Type Culture Collection (ATCC, Manassas, VA 20110, USA) were seeded at a density 350,000 cells per well in a 12 well cell culture plate. Lipofectamine was utilized as a transfection reagent. 25 µL of OPTI-MEM per well and 0.5 µL of lipofectamine per well was used in this transfection process. In order to transfect HEK 293 cells, 0.5 µg of pNL4-3 plasmid was added in each well. After adding the specific quantity of lipofectamine and Opti-MEM to each well, the contents were mixed well by pipetting up and down, and then the solution was incubated for 7 min at room temperature. In order to ensure the success of the transfection process, 0.1 µg of eGFP was also added to each well. Then, the pNL4-3 along with Opti-MEM were also added, and the solution was incubated for 20 min at room temperature. 50 µL of this mixture was added to each well containing the cells. Cell culture media (DMEM + 10% FBS with No Gentamicin) was changed after 24 h. After three days of the transfection process, the contents of each well were collected in a 10 ml tube and were spin down at 1000 rpm for 5 min. The supernatant was collected. The cell supernatant contained viral particles. 100 µL supernatant was used as a viral sample.

#### 2.2.5. Immobilization of Capture Antibody to Beads

Dynabeads (M-280) streptavidin-coated magnetic microbeads were conjugated with biotinylated anti-HIV-1 gp120 antibody for visualization of the captured HIV-1 virions inside a microchip. Figure S1 demonstrates the process of antibody conjugation to microbeads. Dynabeads are critical for this application due to their high refractive index. The refractive index is an intrinsic material property of microbeads that provides invaluable information for various imaging and biosensing applications [36]. Dynabeads have a higher refractive index than silica microbeads as per the company’s provided information. As a result of the higher value of the refractive index, Dynabeads have a characteristic advantage of better detection.

In order to attach the antibody to these beads, we have utilized the manufacturer protocol. Initially, the stock solution containing the beads was vortexed for 30 s. 100 μL of the microbeads were collected in an Eppendorf Protein LoBind tube (14-282-304, Fisher Scientific). One mL of washing buffer (PBS with pH7.4) was added to the microbeads. The vial containing the beads and washing buffer was placed in the close vicinity of a permanent external magnet for one minute. The microbeads were attracted to the permanent magnet and they formed a pellet. The supernatant was removed with the help of a pipette. The microbeads were resuspended in the 100 microliters washing buffer. This washing process was repeated twice. As per the manufacturer’s specifications, 10 μg of biotinylated anti-HIV1 gp120 antibody was added to these washed microbeads. The sample was incubated on a shaker (15 RPM) for 30 min at room temperature. As a result of the very strong interaction between biotin and streptavidin, antibody coating of the Dyna beads was accomplished. The antibody immobilized magnetic beads were collected with the help of an external permanent magnet, and the supernatant was discarded. The antibody-conjugated magnetic microbeads were washed three times. The unoccupied sites of these antibody-coated beads were blocked with 4% BSA solution. This blocking was done overnight at 4 °C on a shaker. The microbeads were collected with the help of a permanent magnet and washed again with PBS. The blocked antibody-coated magnetic microbeads were resuspended in PBS solution and stored at 4 °C for further downstream applications.

#### 2.2.6. HIV-1 Viral RNA Extraction, cDNA Synthesis, and Quantification Using Real-Time qPCR

Viral RNA was extracted using TRIzol reagent (Invitrogen, Carlsbad, CA, USA) according to the manufacturer’s protocol. 11 µL of RNA (out of total 50 µL of RNA) was utilized for the cDNA synthesis with SuperScript Reverse Transcriptase system (Invitrogen). Two µL of 1:3 diluted synthesized cDNA was used for RT-qPCR analysis using Green-2-Go qPCR master mix (Bio Basic, New York, NY, USA) with primers Ex8_5a (TTGCTCAATGCCACAGCCAT) and Ex8_3a (TTTGACCACTTGCCACCCAT). pNL4-3 plasmid DNA was utilized as a standard. PCR amplifications were performed on an AriaMx Real-time PCR System (Agilent, Santa Clara, CA, USA) for thermal cycling and SYBR detection with two replicates for each sample. The quantification of the viral samples was determined by comparing the cycle threshold (Cq) value based on the standard curve generated by the known pnL4-3 plasmid DNA samples amount.

#### 2.2.7. Software

We implemented the ASMCount algorithm using the C++ computational wave optics library [37], OpenCV, and the NumPy numerical processing library in Python. Images were processed in two stages. First, we used a C++ program to obtain inverse diffracted images. These images were fed into a second program containing the counting algorithms to obtain the final counts. The inverse diffraction software was implemented in C++ for performance reasons, but this was not necessary for the counting software. The counting algorithm was implemented using the Python OpenCV bindings. The inverse diffraction algorithm requires one parameter: the distance along which to propagate the diffraction patterns. Ideally, this value could be determined directly from the distance between the sensor and the slide. While this distance produces reasonable results, the best results are obtained when the distance is tuned for best performance since the true distance is difficult to measure precisely. It is sufficient to use the same configuration for all images taken at one time. We performed grid-search to find distances that produced the sharpest images for the counting software, but one may also auto-tune the distance parameter by maximizing the Laplacian sharpness [38]. The developed algorithms are computationally efficient enough to provide results in a few seconds.

## 3. Results

### 3.1. Development of a Lensless Imaging System

We have developed a portable imaging setup using a CMOS sensor to achieve a wide field of view imaging (Figure 1a–g). Lensless imaging setup enables wide-field imaging of the microbeads captured inside the microchip. When the emitted light passes through the captured entities diffraction patterns are recorded using the CMOS image sensor. The output of the sensor is then processed by an image processing algorithm that is used to invert the diffraction patterns into images of the particles on the microfluidic chip surface. This image is then passed to a particle counting algorithm that quantifies the microbeads and hence determines the viral load.

In an initial calibration test, we imaged different-sized microbeads to establish the detection limits of our device. We used a range of differently sized NIST-traceable microbeads (3, 5 and 7 µm). Diffraction patterns of these microbeads were recorded using the setup we developed and processed with custom-made software termed as ASMCount to obtain particle counts (Figures S2–S4). Images of the microbeads can be reconstructed by propagating the diffraction patterns from the CMOS plane to the object plane using the angular spectrum method (ASM). ASM is a technique derived from Fourier optics utilized to propagate fields from measured values in one plane to unknown values in a parallel plane [39,40,41,42]. To propagate an optical field from a set of known planar measurements, the ASM begins by applying the 2D spatial Fourier transform [43]. Since the CMOS sensor captures digital images of the sample, we used the 2-dimensional Fast Fourier Transform (2D FFT) on the sensed image, where the sampling rate for the FFT was determined by the physical pixel size of the camera. In the Fourier domain, the propagation operation becomes a straightforward constant-gain linear-phase filter operation. Therefore, by performing a phase shift in the frequency domain, the ASM propagates the optical field from the CMOS sensor to the slide. The amount of phase shift depends on the distance between the source plane and the destination plane for propagation. To obtain the shadows of the microbeads, we performed the inverse Fourier transform on the filter output. Dark locations, or low values, in the shifted plane corresponded to the locations of particles on the slide. This process allowed the reliable detection of microbeads of various sizes (3, 5 and 7 μm) (Figures S1–S3), yielding the same microbeads patterns as of a conventional optical microscope for our calibration slides. Although we performed zero-padding of the input image, ringing artifacts and bands at the edges of the propagated result image are still observed. This occurs because the ASM propagation filter is not band-limited; therefore, the inverse transform introduces truncation artifacts. We removed these artifacts before the image was passed to a downstream application that enhances and improves the counting of the microbeads. The output of the ASM program is the magnitude, or light intensity, of the field from the inverse-transformed image. Since the sample was illuminated by an ultraviolet LED through a narrow pinhole and the illumination system was located sufficiently far from the sample, a single plane wave was an excellent approximation for the optical field at the slide. The sides of the lighting chamber were painted matte black to ensure that reflections do not violate the assumptions of this model in practice. Therefore, the field propagates in a single direction, which is parallel to the ASM propagation direction, considerably simplifying the problem. The ASM program requires several key parameters from the physical device design: the LED wavelength, CMOS sensor pixel dimensions, and separating distance between the CMOS sensor and the slide. Due to the small microparticle size, the results are quite sensitive to the separation distance, which can vary even within experiments. We addressed this issue by running the ASM for a range of sensor-plane separation distances and selecting the sharpest images. The ASM program is not as sensitive to the other parameters, which were obtained from the UV and sensor datasheets. We passed the output of the ASM program through an image processing pipeline to count the microbeads and visually enhance the image. We applied Gaussian low-pass filters to the images to remove the variable background and we threshold the image to obtain a binary mask. The threshold value is chosen so that the microbeads, which are darker than the background, are extracted from the rest of the image. In the next step, we determined the contours in the thresholded image using OpenCV, a popular open-source image processing library, to obtain a set of objects in the image. This set of objects contains both the microbeads and noise from the ASM artifacts and debris on the slide. To remove false positives and count only the microbeads, we filtered the contours by size and shape. When compared to debris and artifacts, particles are larger, more circular, and more uniform in terms of the object area distribution. Therefore, we removed particles that deviate substantially from a circular shape and the average particle size. Once completed, this process yields an accurate microparticle count and a set of enhanced images that display the microbeads (Figures S1–S3). These results demonstrate the rapid, high throughput imaging of microbeads using the developed lensless imaging platform and the ASMCount software.

### 3.2. Validation of the Microfluidic Chip Capture and Lensless Imaging

As the average size of HIV-1 virions is approximately 145 nm [44,45,46], these viruses cannot be detected without using antibody-conjugated microbeads. For this purpose, we selected 2.8 microns sized streptavidin-coated Dyna beads. These microbeads have a high refractive index, can be easily detected using the lensless imaging setup we developed and can be efficiently coated with the biotinylated anti-HIV1 gp120 antibody. These anti-HIV1 gp120 antibody-conjugated magnetic beads were utilized to detect and quantify the captured viruses to estimate the HIV-1 viral load.

**Figure 2:**
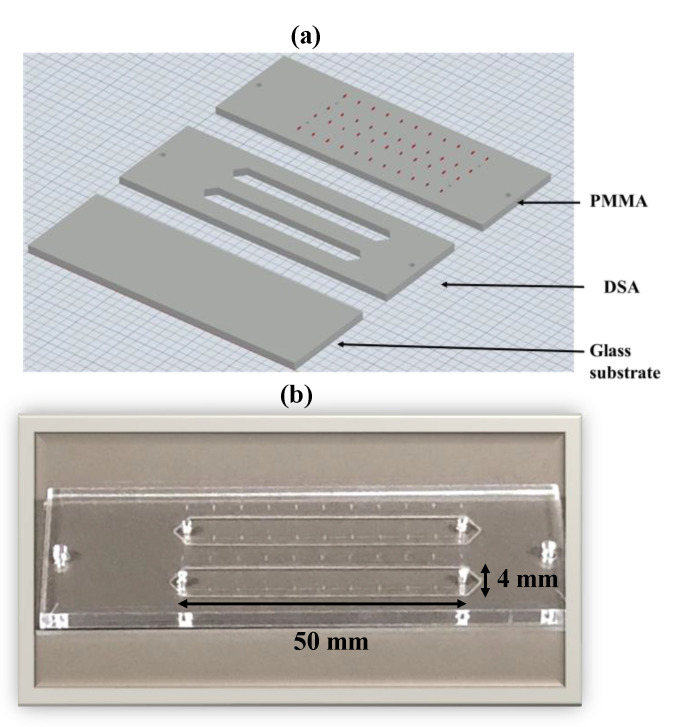
Assembly of microfluidic chip (**a**) Three-Dimensional illustration of the microfluidic device assembled with the antibody-coated glass slide, DSA, and PMMA. (**b**) Photograph of the final assembled microchip.

A microfluidic chip was designed and functionalized using highly specific antibodies for HIV-1 virus capture. The Methods section provides detailed information about the functionalization of glass slides with anti-HIV1 gp120 antibody, which recognizes the viral envelope glycoprotein (gp120) [6,13,47,48,49,50,51]. Briefly, the bottom glass substrate of the microchip was coated with silane-polyethylene glycol (PEG)-thiol. Then, the oxidized antibody was immobilized to the glass slide using a cross-linker 3-(2-pyridyldithio) propionyl hydrazide (PDPH). The surface chemistry employed for coating antibodies to the glass slide was purposely chosen due to its high specificity and negligible non-specific binding. Figure 2a illustrates the chip assembling process that utilizes poly-methyl methacrylate (PMMA), double sided adhesive (DSA) tape and an antibody-conjugated glass slide. The final assembled microfluidic chip was coated with anti-HIV1 gp120 antibodies (Figure 2b).

**Figure 3:**
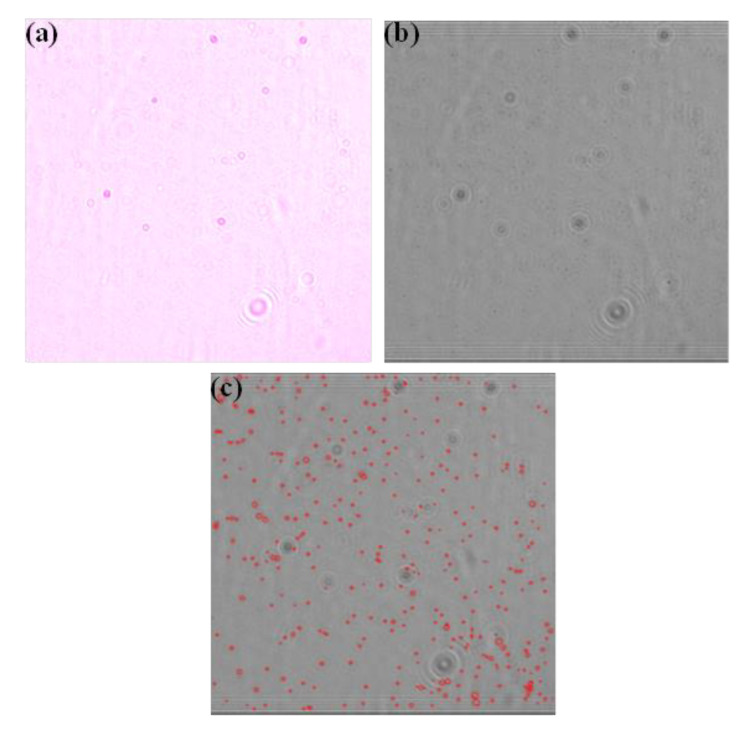
HIV-1 viral particles’ quantification using the microfluidic chip coupled with the lensless imaging method (**a**) Diffraction patterns of 2.8 microns sized microbeads bound to the HIV-1 virions captured inside antibody-coated microchip (**b**) Reverse-diffracted image of the diffracted patterns. (**c**) The quantification process of the 2.8 microns sized microbeads using computational analysis (Count = 350).

The viral sample was injected into the microchannel and incubated onto the anti-gp120 antibody functionalized surface to capture the virions. After the virions were captured, anti-gp120 antibody-coated microbeads were utilized to increase visualization and detection of the captured viruses. The anti-HIV1 gp120 coated microbeads were injected and incubated inside the microfluidic chip for 10 min. The slow injection of the antibody-coated beads resulted in the formation of virus-bead complexes bound to the microchip surface. Each antibody-coated magnetic bead specifically binds to one virus only. If two virions are bound on the surface very nearby, then one bead can attach with more than one virus too, but we do not foresee it as a problem considering the large surface area available for each virus to bind. After this step, the microfluidic channel was washed with an isotonic solution to ensure the removal of unbound microbeads and other debris. The microchips were then placed on top of the CMOS imager, and the diffraction patterns were recorded by the sensor. Each disposable one-time usable microchip contained engraved markings over its topmost PMMA layer to facilitate the recording of diffraction patterns using the CMOS image sensor. The whole microfluidic channel was divided into ten subsections. The surface area of each subsection was purposely chosen (20 mm^2^) to reliably record the images of captured beads using the CMOS imager. Ten consecutive images of each microfluidic channel were recorded. After pre-processing, the diffraction patterns (Figure 3a) recorded by the CMOS image sensor were reverse diffracted using the angular spectrum method (ASM). Those reverse-diffracted images (Figure 3b) were passed to the image processing pipeline to count the microbeads (Figure 3c). Three sections of the microchip near the inlet, center, and outlet were imaged using an optical microscope. Five consecutive images were recorded in each section to confirm the presence of microbeads. The images confirmed that the diffraction patterns obtained using the lensless imaging setup detected only microbeads.

**Figure 4:**
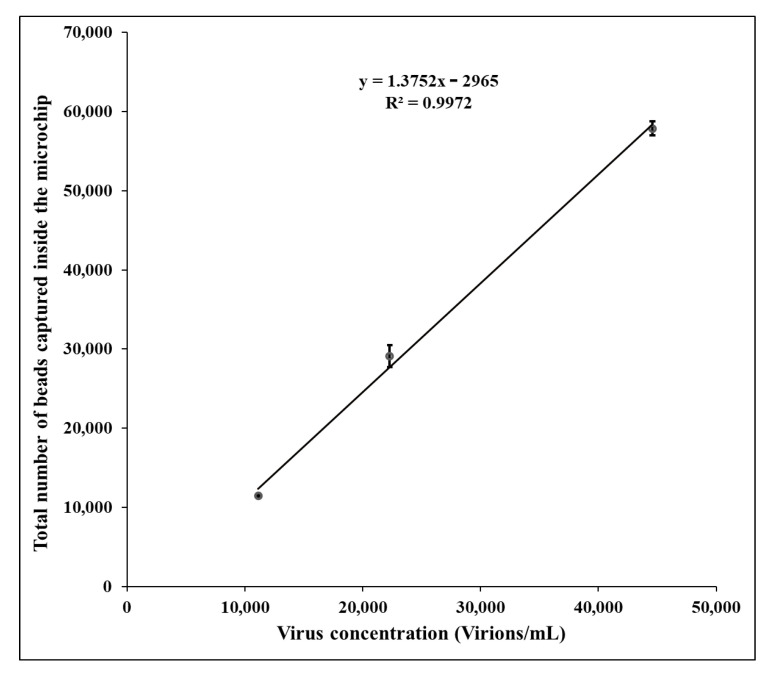
Comparison of quantification results obtained by the microfluidic chip coupled with the lensless imaging method and RT-qPCR. (Each sample was run in duplicate).

We tested the sensitivity and reliability of the microfluidic chip HIV-1 capture coupled with the lensless imaging system utilizing an HIV-1 preparation of known titer. Three samples were tested (undiluted, 1:2 dilution, and 1:4 dilution). The viral load in the undiluted viral sample was determined by RT-qPCR at 44,585 virions per mL. The microfluidic chip–lensless imaging analysis showed 57,880 virions/mL in the undiluted sample. Consistently, the 1:2 and 1:4 dilutions were quantified as 22,293 and 11,146 virions/mL by RT-qPCR, respectively, and 29,090 and 11,430 virions/mL, respectively, utilizing the microfluidic chip coupled with the lensless imaging method (Figure 4). Thus, showing the high reliability and accuracy of the data generated by the lensless imaging method when compared to the standard RT-qPCR.

To further demonstrate the viral load quantification capability of the microfluidic chip with the lensless imaging method, we tested five different HIV-1 viral samples. The viral count obtained by this method was compared with the gold standard RT-qPCR. The quantification results of both methods are presented in Table 1. It clearly indicates that this developed method can be utilized to determine the HIV-1 viral load in POC settings.

In all these samples, the microchip-based analysis showed a 10–25% higher viral load when compared to the RT-qPCR data. This is likely due to the loss of RNA experienced during the RNA extraction protocol required for the RT-qPCR assay. Although the TRIzol extraction protocol we utilized is highly quantitative, a loss of 10–25% of the starting material is expected.

In the RT-qPCR method, during the phase separations, 10–25% of the aqueous phase is lost to prevent DNA contaminations. It is mandatory not to disturb the interface between the aqueous and organic layers. As a result, there is an obvious loss of 10–25% of the starting material. There is no such limitation in the case of the microfluidic chip-based method. Thus, the results we obtained utilizing the microchip-lensless imaging method suggest higher reliability of this assay when compared to an RT-qPCR method requiring an RNA extraction step.

In all our assays, we also run a parallel analysis of a no-virus control sample. Overall, we detected an average background of approximately 2400 (±114.4) microbeads bound in the microchip. The adventitious binding of microbeads on the chip surface was minimized using silane-polyethylene glycol-thiol based surface chemistry [52,53]. The surface chemistry using NeutrAvidin on chemically activated surface including N-g-Maleimidobutyryloxy succinimide ester (GMBS) and 3-Mercaptopropyl trimethoxysilane (3-MPS) modifications results in a significant amount of non-specific binding (data not shown here). This non-specific binding could be further minimized using silane-polyethylene glycol-thiol based surface chemistry for the attachment of anti-HIV1 gp120 antibody for the capture of HIV-1 viral particles. The sensing range of this assay is 11,146 to 44,585 virions per mL. The developed assay has a limit of detection of 1569 virions per mL.

## 4. Discussion and Conclusions

We have developed a portable lensless imaging setup that can be utilized with a disposable antibody-coated microchip for the rapid quantification of HIV-1 viral load. This setup is a cost-effective solution for early-stage HIV-1 diagnosis, combining microfluidic technology with wide field-of-view lensless imaging. HIV-1 viral particles are captured on the surface of a microfluidic chip functionalized with anti-HIV1 gp120 antibodies. Microbeads, which are also functionalized with anti-HIV1 gp120 antibodies, are then utilized to identify the captured virions. The virion bound microbead images can be recorded using a lensless imaging setup. Our microchip-based approach is inherently more accurate when compared to the gold-standard RT-qPCR method. The increased accuracy is because of quantification biases due to the RNA degradation, extraction and amplification inhibition are significantly reduced in immunoassays involving the capture of intact virions [54].

Our imaging setup presents several advantages that make it well-suited for POC operating conditions. For instance, the image sensor supports high throughput quantification; the LED and sensing components are low-wattage; are compatible with most commercially available power supplies and, due to the lack of lenses and other optical equipment, the platform is neither fragile nor bulky, it is easy to transport and use. Produced at scale, the overall cost of the assay would be less than $15, including the cost of the microchip and biological/chemical reagents (Table S1).

The setup we developed is well-suited for the detection of the HIV-1 virus at the acute infection stage. Although HIV-1 viral load is at peak at this stage (10^6^–10^8^ virus copies per mL) [12,55,56,57,58], antibody-based assays cannot detect the presence of viruses because antibodies appear 3–6 weeks after the initial infection [59]. People with acute HIV-1 infection are unaware of their disease status, are highly infectious due to higher viral load, and, as a result, can significantly contribute to the spread of HIV-1 [60]. Thus, identifying individuals in the early stages of acute HIV-1 infection is essential to stop the further transmission of the virus. The microfluidic chip coupled with the lensless imaging method is an assay well suited for the detection of acute HIV-1 infection at POC settings and in areas with limited resources. The current LOD of this developed microfluidic device is suitable for the detection of acute HIV-1 infection where viral load is very high. In future, we intend to further improve the sensitivity of the assay using a virus enrichment step.

When compared to the conventional microscope-based imaging platforms, the lensless setup we developed is portable, simple, light-weight [61], and offers a large field-of-view (FOV) that enables high throughput medical diagnostics [62]. Such a device has the potential to revolutionize the HIV-1 diagnostic arena because it requires a minimal amount of sample and limited biological/chemical reagents with a total assay time of around 100 min. Finally, since the use of a fully automated setup can significantly enhance throughput and reliability, we are planning to automate the manual processing steps using microfluidic chambers and valves. As an added advantage, this platform technology can be easily adapted and applied to the quantification of other emerging pathogens like SARS-CoV-2.

## Figures and Tables

**Table 1:** Comparison of quantification results obtained by the microfluidic chip coupled with the lensless imaging and the gold standard RT-qPCR for five different viral preparations.

Microfluidic Chip Coupled with the Lensless Imaging Method (Virions/mL)	RT-qPCR Technique (Virions/mL)	Percentage Difference between Readings of Microfluidic Chip Coupled with Lensless Imaging Method and RT-qPCR Technique
1,217,000	921,000	24.32
598,000	461,000	22.91
409,000	307,000	24.94
222,000	186,000	16.22
212,000	182,000	14.15

## Data Availability

Not applicable.

## Supplementary Materials

The following are available online at https://www.mdpi.com/1424-8220/21/5/1819/s1, Table S1: The overall costs of portable imaging setup, microchip, and assay. Figure S1: Illustration of anti-HIV1 gp120 antibody conjugation to streptavidin-coated Dyna beads. Figure S2: Image acquisition and subsequent quantification of 3 microns sized microparticles using developed method. Figure S3: Image acquisition and subsequent quantification of 5 microns sized microparticles using developed method Figure S4: Image acquisition and subsequent quantification of 7 microns sized microparticles using developed method.
