# Supplementary material for: Development of a Point-of-Care Assay for HIV-1 Viral Load Using Higher Refractive Index Antibody-Coated Microbeads
Source: Sensors (Basel). 2021 Mar 5;21(5):1819. doi: 10.3390/s21051819 (PMC7961362; doi:10.3390/s21051819)
Supplement: Supplementary file 1 [file sensors-21-01819-s001.pdf]

sensors

Article

Development of a Point-of-Care Assay for HIV-1 Viral Load Using Higher Refractive Index Antibody-Coated Microbeads

Mazhar Sher [1,2], Benjamin Coleman [3], Massimo Caputi [4] and Waseem Asghar [1,2,5,*]

1 Asghar-Lab, Micro and Nanotechnology in Medicine, College of Engineering and Computer Science, Boca Raton, FL 33431, USA; msher2015@fau.edu
2 Department of Computer & Electrical Engineering and Computer Science, Florida Atlantic University, Boca Raton, FL 33431, USA
3 Department of Electrical and Computer Engineering, Rice University, 6100 Main Street, Houston, TX 77005, USA; ben.coleman@rice.edu
4 Charles E. Schmidt College of Medicine, Florida Atlantic University, Boca Raton, FL 33431, USA; mcaputi@fau.edu
5 Department of Biological Sciences (Courtesy Appointment), Florida Atlantic University, Boca Raton, FL 33431, USA
* Correspondence: wasghar@fau.edu

Citation: Sher, M.; Coleman, B.; Caputi, M.; Asghar, W. Development of a Point-of-Care Assay for HIV-1 Viral Load Using Higher Refractive Index Antibody-Coated Microbeads. Sensors 2021, 21, 1819. https://doi.org/10.3390/s21051819

Academic Editor:

Received: 30 January 2021
Accepted: 28 February 2021
Published: 5 March 2021

Publisher's Note: MDPI stays neutral with regard to jurisdictional claims in published maps and institutional affiliations.

Copyright: © 2021 by the authors. Submitted for possible open access publication under the terms and conditions of the Creative Commons Attribution (CC BY) license (http://creativecommons.org/licenses/by/4.0/).

Table S1. The overall costs of portable imaging setup, microchip, and assay.

Lensless Imaging Platform	
**Components**	**Cost ($)**
100 µm pinhole	67.50
Right angle USB 3.0	6.99
LED	164.48
LED driver	323.55
Variable Current supply	30
3D printer filament	2
UI-3592 LE Camera	518
**Total cost**	1112.52
**Automation Platform**	
**Elements of microchip**	**Cost ($)**
Poly (methyl methacrylate) (PMMA)	0.1
Optically clear Double-Sided Adhesive Tape (DSA)	0.1
**Total cost**	0.2
**Biological Reagents Per Test**	
**Reagents**	**Cost ($)**
ab85054 Goat polyclonal to HIV1 gp120 (ab85054)	13.66
Goat polyclonal to HIV1 gp120 (Biotin) (ab53937)	0.1366
M-280 Streptavidin microparticles	0.065
**Total cost**	13.8616

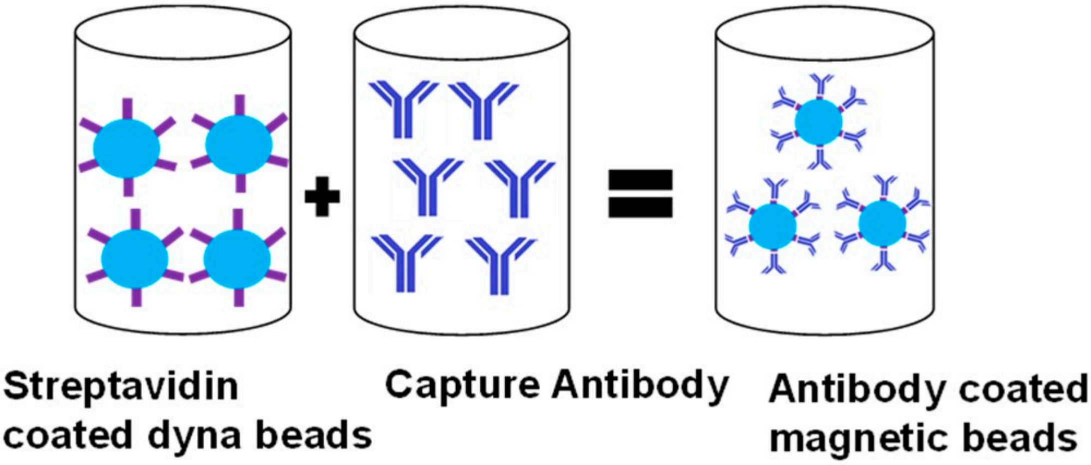

Figure S1. Illustration of anti-HIV1 gp120 antibody conjugation to streptavidin-coated Dyna beads.

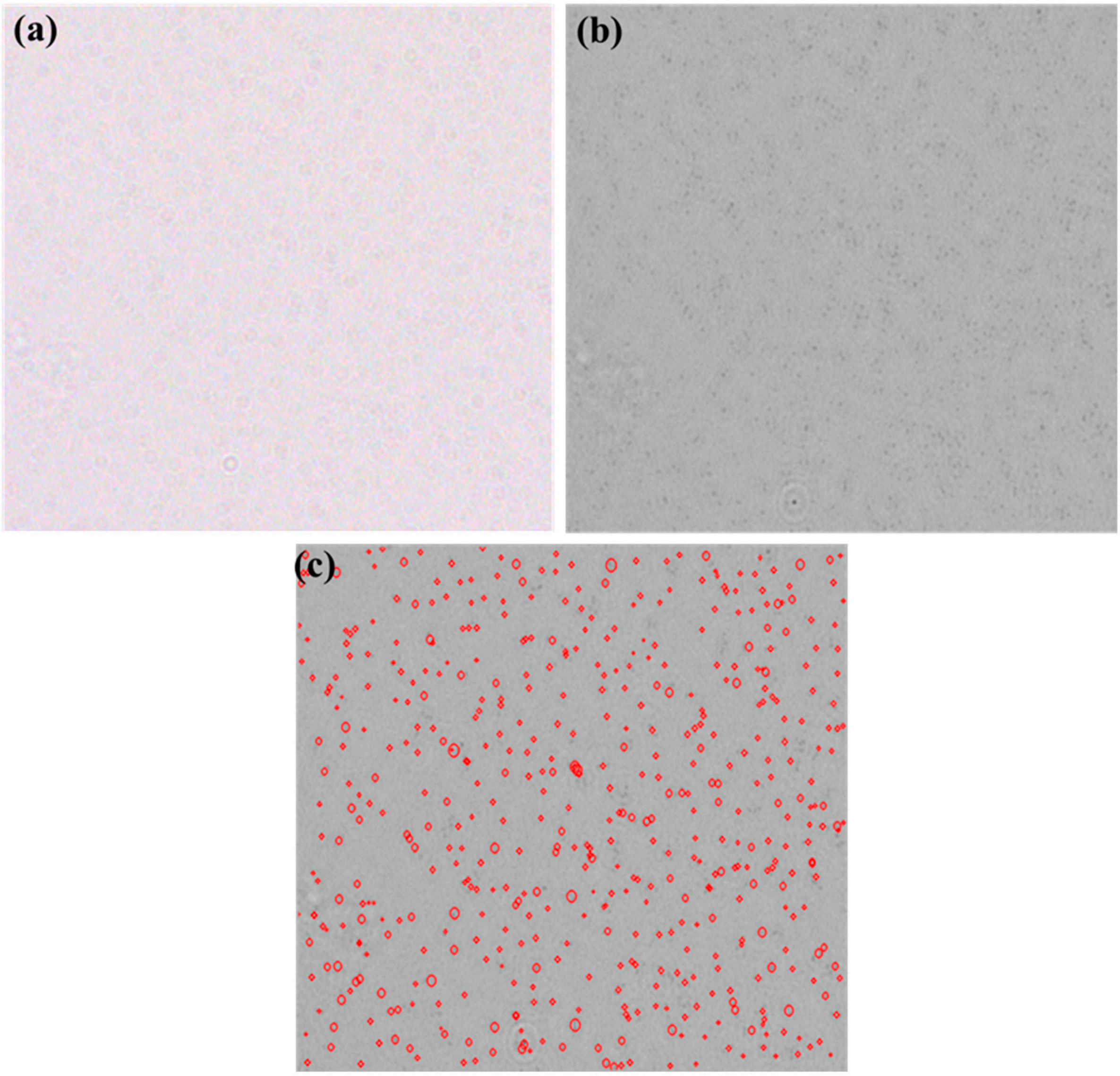

Figure S2. Image acquisition and subsequent quantification of 3 microns sized microparticles using developed method (**a**) Diffraction patterns of 3 microns sized bead (**b**) Reverse-diffracted image of diffraction patterns. (**c**) The quantification of the 3 microns sized particles by a computer program (Count result = 510 microparticles).

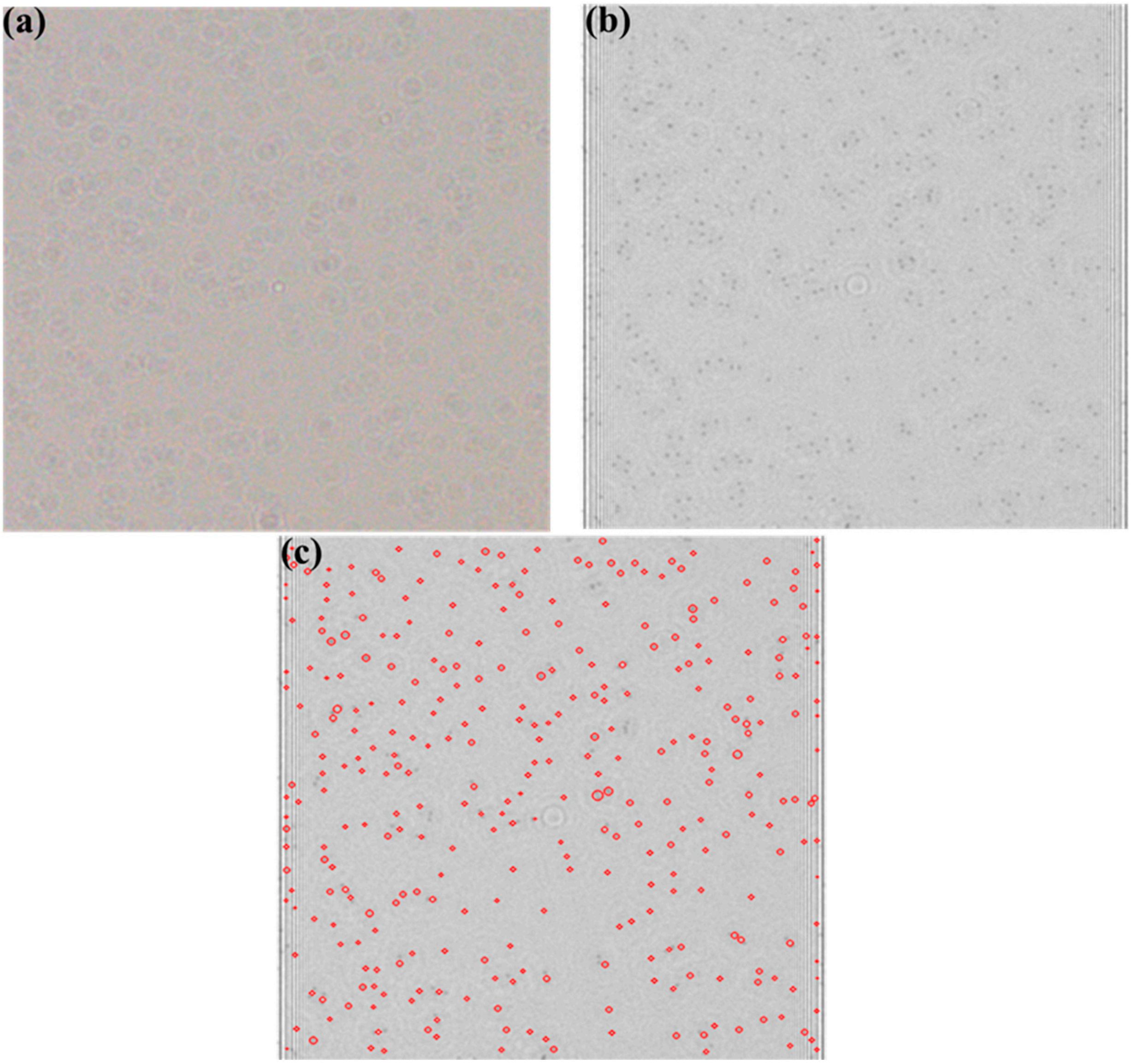

Figure S3. Image acquisition and subsequent quantification of 5 microns sized microparticles using developed method (**a**) Diffraction patterns of 5 microns sized microparticles (**b**) Reverse-diffracted image of diffraction patterns. (**c**) The quantification of the 5 microns sized particles by a computer program (Count result = 325 microparticles).

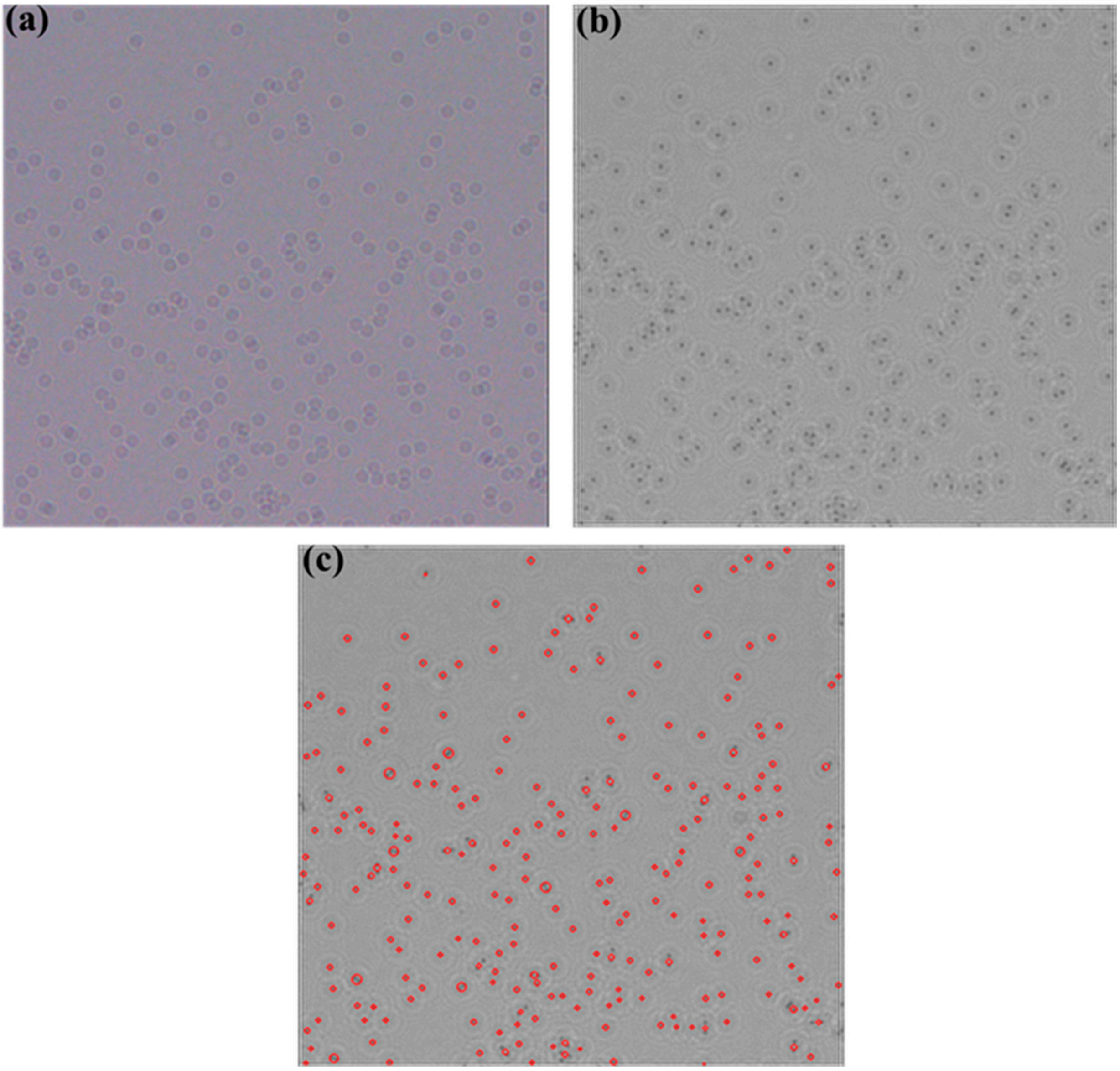

Figure S4. Image acquisition and subsequent quantification of 7 microns sized microparticles using developed method (**a**) Diffraction patterns of 7 microns sized microparticles (**b**) Reverse-diffracted image of the microparticles. (**c**) The quantification of the 7 microns sized particles by a computer program (Count result = 239 microparticles).
